# A new subscapular sling operation to stabilize the shoulder. A cadaver study

**DOI:** 10.1186/s40634-015-0028-y

**Published:** 2015-06-02

**Authors:** P J Klungsøyr, F Guldal, T Vagstad, J A Klungsøyr

**Affiliations:** Orthopaedic Department. Aalesund Hospital, Aalesund, Norway

**Keywords:** Shoulder instability, Arthroscopic sling procedure, Hamstring graft, Subscapularis tendon, Cadaver study

## Abstract

**Introduction:**

A new arthroscopic shoulder stabilisation procedure is proposed, which for some patients could be an alternative to the arthroscopic Latarjet procedure.

**Methods:**

The objective was to stabilize the shoulder by making a sling around the subscapularis tendon, using a hamstring graft and enhancing the anterior rim of the glenoid with the same graft. The anatomical feasibility of the surgical procedure was tested to establish the surgical method.

**Results:**

Four surgeons performed the surgery on six cadavers. After the surgery the cadavers were dissected to visualize the result. The sling was placed according to the intention and the nerves in the area (axillary and musculocutaneus) were not at risk, nor had they altered position during the procedure.

**Conclusion:**

The procedure is technically feasible and the risk of complications seems low. This procedure could be an alternative to the Latarjet procedure and to other operations used for anterior instability of the shoulder. A biomechanical study will be performed as the next stage of the development.

**Clinical relevance:**

This procedure could be an alternative to the Latarjet procedure and to other operations used for anterior instability of the shoulder.

**Trial registration:**

2012/1978/REK sør-øst

## Background

The Latarjet procedure for anterior shoulder instability has today regained popularity. The main indication is instability with a glenoid bone defect, but it is increasingly used as a revision procedure following previous surgery and as the primary procedure in cases with weak anterior structures (Blomquist et al. 2012). The Latarjet operation yields good results, but also has complications (Hovelius et al. 2004, Shah et al. 2012, Griesser et al. 2013). It is currently the first choice for some shoulder surgeons. The Latarjet procedure is considered technically demanding, especially when performed arthroscopically, but even so it is routinely done arthroscopically in some centres (Lafosse et al. 2010, Shah et al. 2012). The learning curve for the arthroscopic operation is long, with significant reported and unreported complications (Butt and Charalambous 2013). Gracitelli et al. (2013) reported results of arthroscopic Latarjet procedure in cadavers in Brazil and found a high risk of complications. If the Latarjet operation fails, a revision procedure might be difficult because of the distorted anatomy and the increased risk of nerve complications. In this regard, the musculocutaneus nerve is at risk, since its position is altered during the operation when the conjoined tendon is pulled inferiorly (Freehill et al. 2013).

In this study, the objective was to develop a procedure that could be an alternative to the Latarjet procedure. The sling effect of the Latarjet around the inferior part of the subscapularis tendon and capsule hinders the anterior translation of the humeral head. In addition, the fixation of the coracoid bone onto the anterior rim of glenoid increases the glenoid size and thus increases the stability. The dynamic sling effect in Latarjet is described in biomechanical testing by Giles et al (2013). The effect seems to be enhanced by the coracoid bone block. The effect of the bone block has also been shown by Yamamoto et al. (2009). Wellmann et al. (2012) showed that the Latarjet procedure will lose anterior stability if the subscapularis tendon is torn.

The Resch method is theoretically an extra capsular tightening of the anterior capsule. To achieve this, Resch used a special portal going through the muscle belly of the subscapularis muscle, using a slalom technique to avoid injuring the conjoined tendon and the musculocutaneus nerve Resch.H et al. (1996). Even though the aim of Resch was not to do a subscapular tenodesis, we believe that the insertion of tacks may cause a partial tenodesis or sling effect. The posterior tendinous sheath of the subscapularis muscle and the fibrous structures around it are strongly connected to the anterior capsule and thus pulled towards the anterior rim of glenoid in the Resch procedure. When performing the Resch method we observed that the fixation of the plugs resulted in a reduced external rotation that could be partly a result of an effect on the subscapularis muscle. As the structures are gradually stretched out over time, the effect diminishes and this could explain the excellent primary results and the high numbers of late recurrences Cartus et al. (2007).

This study therefore proposes an arthroscopic operation that could be used in patients where the Bankart operation was judged to be insufficient and as a possible alternative to arthroscopic Latarjet. The proposed procedure should have less potential complications and a shorter learning curve. The hypothesis is that the sling effect can be created by a technically less demanding procedure, and can be made in a manner that would remove the need for a bone block.

## Methods

**Fig. 1:**
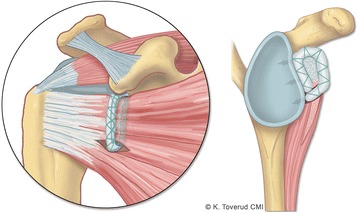
Drawing of the sling

The proposed procedure makes a sling around the upper part of the subscapularis tendon with a hamstring graft and at the same time increases the size of the soft tissue structures anteriorly with the same graft (Fig. 1) to increase the size of the glenoid cup. The increase in soft tissue on the anterior rim of the glenoid, as a widening of the cup, could prevent engagement of Hill-Sachs lesions. The sling would prevent the dislocation of the humeral head through the active and passive function of the subscapularis tendon in the sling. To achieve this, the sling should be placed around the upper part of the tendon and not the inferior part as in Latarjet. By placing the sling in such a manner, the inferior movement could possibly be more restricted. The Latarjet procedure has only one leg of the sling attached to the glenoid and does not, according to Wellmann et al. (2012), hinder the inferior movement. The proposed sling will have two legs and might prevent inferior movement by hindering the subscapularis tendon from sliding inferiorly and thus assisting in keeping the humeral head in place. The hamstring tendons are used as a strong and lasting tissue replacement in many locations, are easy to harvest and do not give much donor site discomfort. The intention was to use the semitendinosus tendon and do the procedure arthroscopically.

After the Regional Ethics Committee gave its approval, the procedure was tested on cadavers at the Smith and Nephew Surgical Skills Centre, York, North Yorkshire, United Kingdom. Four experienced shoulder surgeons used two working stations and six fresh frozen right shoulder cadavers including half of the upper arm. The specimen was fastened to a table with a scapula clamp in a Beach chair position. There was no available equipment for measuring exact movements, any translations or any form for biomechanical testing. The surgeons worked in pairs and rotated so all surgeries were not dependent on one surgeon. The centre provided already harvested hamstring tendons. Standard arthroscopic equipment and Bioraptor 2.9 anchors with two preloaded sutures were used. Three portals were introduced; posterior for the scope, anterior upper for instrumentation and inferior anterior for graft introduction. For the graft portal (3^rd^ portal) an 11 mm portal, and for the upper, a standard 8 mm portal were used. Preoperatively the placement of the inferior anterior portal was discussed. We had experience with the Resch slalom technique and used this experience to go inferior with the portal at the same level as Resch used. By going through the tendinous part of the subscapularis tendon the risk of injuring the musculocutaneus nerve would be reduced. Since we intended to make a split mainly in the tendinous part and not in the muscular part of subscapularis tendon, the slalom technique was not used. The graft was doubled.

**Fig. 2:**
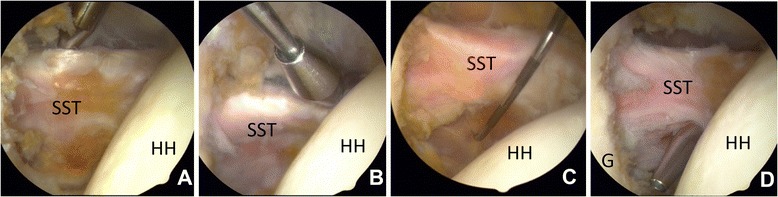
Arthroscopic pictures showing the preparation of the subscapularis tendon. **a**: Dissecting the anterior surface of the tendon from the upper portal. **b**: From the inferior portal. **c**: The point of entry for the slit in the subscapularis tendon from inside. **d**: The trocar advanced through the slit. Abbreviations: SST: Subscapularis tendon. HH: Humeral head. G: Glenoid. P: Portal

**Fig. 3:**
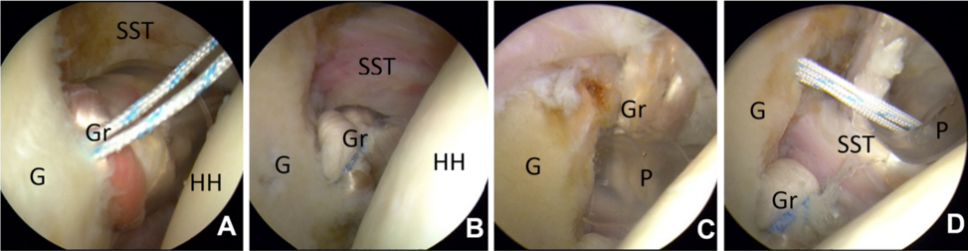
Arthroscopic pictures showing the introduction of the graft. **a**: The graft has been pulled in towards the glenoid anchor through the portal. **b**: The portal is withdrawn. **c** and **d**: The portal is placed anterior and superior to the subscapularis tendon and the graft is pulled out through the upper portal. Abbreviations: SST: Subscapularis tendon. HH: Humeral head. G: Glenoid. Gr: Graft. P: Portal

**Fig. 4:**
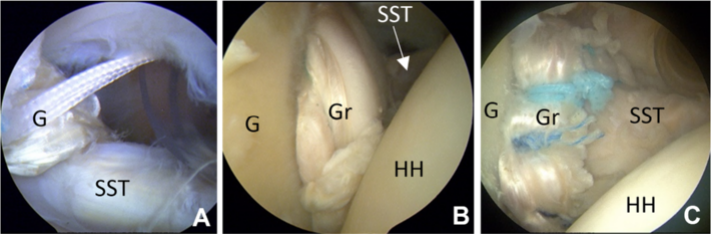
Arthroscopic pictures of the final placement of the graft seen from inside. **a**: The upper leg is fixed to the glenoid with the first suture, the second suture goes into the upper portal. **b** and **c**: The upper part of the graft placed on the anterior ridge of glenoid. Abbreviations: SST: Subscapularis tendon. HH: Humeral head. G: Glenoid. Gr: Graft. P: Portal

After placing the two first portals, the layer anterior to the subscapularis tendon (Fig. 2-a) was opened up. By doing this a small part of the rotator interval was opened. Blunt dissection with a trocar through the upper portal exposed the inferior anterior part of the tendon. Then the inferior anterior portal was introduced. First by using a trocar from outside and with coblation (radiofrequency) from inside. A longitudinal slit in the capsule and subscapularis tendon was made and the portal was introduced into the joint (Fig. 2-b,c and d). The anterior surface of the glenoid neck was cleared by coblation. An anchor with two pairs of sutures was introduced through the 3^rd^ portal on the anterior rim of glenoid between 4 and 5 o’clock. Using a sliding knot, the end of the transplant was pulled in and fixed to the anterior glenoid rim (Fig. 3-a and b). The portal was then partly withdrawn anteriorly to the subscapularis and moved into the joint above the tendon. By the help of an extra suture on the outer end of the transplant it was pulled into the joint above the subscapularis tendon and out through the upper portal (Fig. 3-c). A new anchor was introduced (using the 3^rd^ portal in its new position) at around 2 o’clock and the graft was slightly tightened and sutured to this anchor (Fig. 3-d and 4-a). The rest of the graft was pushed back into the joint and placed along the anterior glenoid rim, sutured with the second suture of the first and second anchor. Additional anchors could be inserted to make an even firmer fixation. As a result the end loop would be placed as an extra tissue wall on glenoid anteriorly, similar to a thick labrum (Fig. 4-b and c).

After the arthroscopic procedure, the shoulder was dissected to study the graft placement and the relation to the main nerves. The placement of the anterior inferior portal was evaluated. The deltoid was dissected away from its upper attachments and then the conjoined tendon was detached and reflected inferiorly so the graft, the slit, the subscapularis muscle-tendon and the actual nerves could be inspected.

## Results

**Fig. 5:**
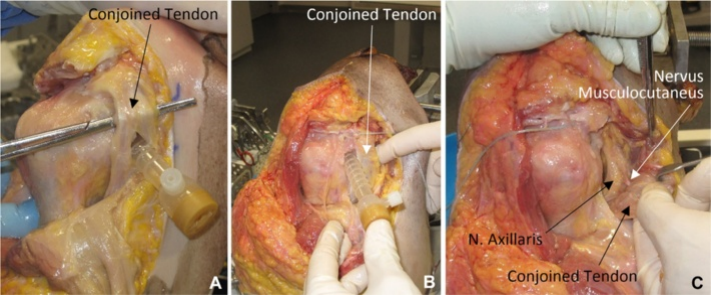
Post-operative pictures. **a**: Portal placed through the conjoined tendon. **b**: Correct portal placement. **c**: The musculocutaneus nerve is seen in the reflected conjoined tendon/muscle. The axillary nerve is seen in the fatty tissue

**Fig. 6:**
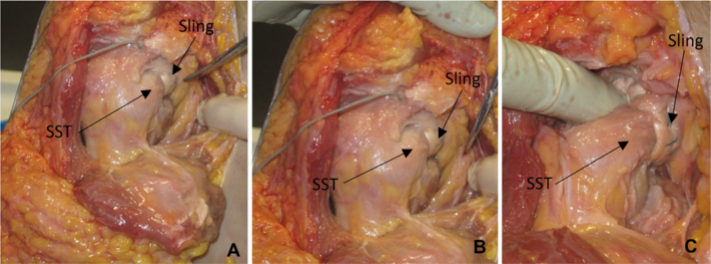
Post-operative pictures: **a** and **b**: Placement of the sling. **c**: Supraspinatus and the anterior part of the inferior capsule is cut. The sling holds the head in the glenoid cavity. Abbreviation: SST: Subscapularis tendon

The procedure was performed in six cadavers with different combinations of surgeons and none encountered any major difficulties in performing the procedure. In the first cadaver, the lower anterior 3^rd^ portal was made through the conjoined tendon (Fig. 5-a). On the following cadavers the skin incision was made 0.5 cm lateral to the line inferior from the tip of coracoid in the line of the upper arm and the portal 90 degrees on the frontal plane, which successfully avoided the conjoined tendon (Fig. 5-b). The distance from the anterior tip of coracoid to the portal was 2 cm in all. In none of the cadavers was the musculocutaneus nerve injured, nor was its position altered. The axillary nerve was dissected and it was not at risk in any of the cadavers (Fig. 5-c). It was not possible to make accurate measurements of the distance from the nerves to the 3^rd^ portal and from the end of the slit to the axillary nerve because of the condition of the tissue, extravasation of fluid and altered consistency of the tissue because of the handling before surgery. Since the axillary nerve crosses on the anterior surface of the subscapularis muscle it could be injured while doing the blunt dissection on the anterior surface of the muscle, but the distance between the medial end of the split in the tendon and the nearest location of the axillary nerve was measured to be at least 1.5 cm on all the cadavers. The width of the subscapularis tendon in the created sling was 1.5 to 2.5 cm. A substantial part of the tendon was thus included in the sling (Fig. 6-a and b). Since the tendon is rather flat the width of the graft inside the sling would also depend on the tension applied to the graft. The measurements were done with the arm tentatively in neutral rotation and in the line of the body. Although a rather short longitudinal split in the subscapularis tendon was made, the movement of the subscapularis tendon inside the sling was visualized arthroscopically.

One of the cadavers was unstable in all directions before the surgery because of a massive rupture of the rotator cuff, but with the subscapularis tendon intact. Prior to the procedure the head was dislocated inferiorly, but after the surgery the humeral head was in place and the joint was stable under quite forceful manual testing. In one of the cadavers with an intact rotator cuff, the upper cuff was released from the humerus, but still the sling kept the head up (Fig. 6-c). Since the set up was not designed for proper testing of the movements, exact measurements cannot be given. As far as could be tested manually, the external/internal rotation, flexion and abduction were not restricted in any of the cadavers after the operation. In all the cadavers the outward rotation was estimated as not less than 45 degrees. We did not dissect the suprascapular nerve posteriorly to the joint because we had not introduced any instruments or placed any anchors/screws which could possibly cause injury to this nerve. This complication is described by Gracitelli et al. (2013).

## Discussion

The procedure could be performed without any major technical difficulties and no nerve injury was observed. All four surgeons did the surgery and there were no differences in performance. To avoid perforating the conjoined tendon we had to alter the placement of the inferior anterior portal, making it slightly more lateral. During the dissection after the arthroscopic procedure, it was noted that neither the musculocutaneous nor the axillary nerves were at risk, nor had they altered position. According to the reports of both open and arthroscopic Latarjet procedure there is always a change in the anatomical position of the musculocutaneus nerve that could represent a danger if a reoperation has to be performed later (Freehill et al. 2013). This will not be the case with the proposed procedure since the conjoined tendon is not being manipulated. During both the open and the arthroscopic Latarjet procedure there is always a manipulation of the area on the anterior surface of the subscapularis muscle close to the axillary nerve and there are also reports of injuries to the nerve (Shah et al 2012). Judged by findings during dissection, there is less risk of axillary nerve involvement in the proposed operation.

The procedure is probably technically easier to perform than the arthroscopic Latarjet. No more difficulties were noted in performing this operation than during an arthroscopic Bankart operation. Problems with bleeding are not generally encountered while clearing the anterior surface of the subscapularis tendon in the open Latarjet operations. By comparing the amount of dissection done in an arthroscopic Latarjet, it is expected that the proposed operation would have a lower risk of bleeding.

Wellmann et al (2012) showed that the sling effect of Latarjet hinders anterior movement, but the sling would not hinder inferior movement. Dines et al. (2013) also showed that the Latarjet procedure hindered the anterior translation of the humeral head while preserving the ROM. Our hypothesis is that a sling with two legs attached to the glenoid would better hinder the inferior movement as compared with the Latarjet sling with only one leg fixed to glenoid rim. The two legged sling could hinder the subscapularis muscle from being pulled inferiorly and since the subscapular muscle is a very important active stabiliser of the joint, this would hinder inferior movement and give additional stabilisation. The cadaver that had an intact subscapularis tendon with a massive cuff rupture was stabilized with the sling. The sling in that particular cadaver provided passive stabilisation so it may be that the upper sling could act as a stabiliser by both passive and active forces. Additional biomechanical testing must be performed. In another of the cadavers, the superior rotator cuff was completely severed, but it did not seem to reduce the stability when the sling was in place.

It is possible that the sling could cause degeneration and rupture of the subscapularis tendon and there could be atrophy of the muscle. Open operations with an L-shaped incision in the subscapular muscle will, according to some publications, lead to reduction in the strength and some atrophy of the subscapularis muscle (Paladini et al. 2012), but the present study suggests this problem is at least very much reduced when a subscapularis split has been made. Hiemstra et al. (2008) showed there was no difference in subscapularis function between open and arthroscopic stabilisation in a tendon splitting approach. Strength deficits existed in both the arthroscopic and the open groups when compared to the contralateral limb. We are not aware of any publication that has shown rupture of the inferior part of subscapularis muscle after Latarjet, where only a split has been made. According to Hinton et al. (1994) the insertion part of the subscapular muscle differs in the sense that the superior 60 % consists of tendon and the inferior 40 % mainly of muscle. It was also observed during dissection that there is gradual transition with increasing muscle and decreasing tendon structure moving inferiorly. Degeneration and rupture generally comes in the tendinous part of a muscle which could indicate that an upper sling as we made, could be more vulnerable for degenerations than an inferior sling. We are not aware of any literature discussing this problem in depth. Would our sling cause degeneration and eventually rupture of the subscapularis tendon? The insertion of the tendon was not injured and the sling should not strangulate the tendon. The sling will, according to our thesis, provide dynamic stability in abduction and external rotation through the subscapularis muscle function, but otherwise there would probably not be any tension on the tendon.

A systematic review by Griesser et al. (2013) of multiple medical databases included studies reporting outcomes with complication and reoperation rates following original or modified versions of the Bristow or Latarjet. The total complication rate was 30 %. Recurrent anterior dislocation and subluxation rates were 2.9 % and 5.8 %, respectively. The reoperation rate was 7 %. Mild loss of external rotation was common. Reoperation rates were lower following all-arthroscopic techniques, but there was a greater loss of postoperative external rotation with all-arthroscopic surgery. We cannot see that the proposed upper sling should cause more reduction in the external rotation than a Latarjet procedure. In some patients probably some reduction could be desirable to hinder instability.

Late mobilisation can cause reduced strength in internal rotation and thus early mobilization could be an advantage. To maintain the split in the tendon after the operation it may be essential to start early exercises. In Latarjet a slit is also made and any healing of that slit seems to be of less concern. Early mobilisation could affect the healing of the graft towards the anterior glenoid. This has to be considered before any in vivo operation. Suture anchors have achieved good healing between the capsule and glenoid in other shoulder operations. Post-operative rehabilitation would be similar to standard arthroscopic Bankart operations. In that case this operation should not cause more external rotation lag and subscapularis atrophy than the Latarjet procedure, except for the possibility that the upper sling in itself could cause more degeneration than the inferior sling. The labrum and capsule could also be included in the graft on the anterior rim of glenoid in the same way as Latarjet is often combined with a Bankart procedure.

This is an anatomic study of a new procedure. No biomechanical analysis has been performed. We had planned to do a second alternative of this operation by introducing a bone block with the tendon and fixate it to the lower anterior part of glenoid with sutures or screws and then proceed with the sling. This is possible through the same 11 mm portal. The bone block could be harvested from the tibia together with the tendon graft. To get enough experience with the present operation, it was decided not to do the second alternative. Giles et al. (2013) tested the importance of a sling on the inferior part of the tendon and capsule and found that the additional bone was important when there was a bone defect. The bone defect was quite substantial in their trial. It is possible that a sling around the upper and middle part of the tendon has a higher stability and could give good results without a bone block, since the sling around the upper part might hinder the anterior and inferior translation more effectively. Biomechanical testing is needed to establish this.

Today the Latarjet procedure is used in many hospitals as the primary operation when the anterior structures are weak and not fit for a Bankart operation. This tendency can probably be explained by the disappointing results of the Bankart operation in young patients (Blomquist et al.^2012^). Our sling could be a possible alternative in younger patients, and if it fails one still has the possibility of using the Latarjet procedure without any expected difficulties caused by the primary surgery. Cosmetically the proposed operation would give a better result, since the coracoid is not touched.

## Conclusion

A tendon sling was made around the subscapularis tendon in an arthroscopic procedure on cadavers to prevent anterior instability in the shoulder. As part of this procedure a tissue wall was created anterior to the glenoid that also may help to prevent dislocation. The risk of injuring the two main nerves seems less than in the Latarjet procedure and possible future reoperation will probably have a lower risk of complications since the anatomy is not altered. From a technical perspective, this procedure could be performed safely in vivo. To know the actual effect of the sling, further testing in a biomechanical laboratory is needed, especially in cases with glenoid bone loss. The proposed procedure was feasible in cadavers and technically not demanding.

### Clinical relevance

This procedure could probably be used on patients with a weak anterior capsule even though arthroscopic Bankart will still be widely used. In young patients with less bone loss, where the tendency in some hospitals today is to perform the Latarjet procedure, the proposed procedure could be an alternative. Whether it is applicable in cases with or without bone loss needs to be tested biomechanically. If a bone block is needed, this could be done by harvesting a bone block from the tibia, keeping the original attachment of the hamstring tendon, and doing the identical operation with the additional bone fixation.
